# Prevalence of psychoactive substance use among medical students in the EMRO region

**DOI:** 10.4102/jphia.v16i1.1389

**Published:** 2025-09-18

**Authors:** Nada Bennani Mechita, Anas Ahmed Mountassir, Sara Messaoud, Karim Sbai Idrissi, Hafid Hachri, Khalid Saeed, Rachid Razine, Majdouline Obtel

**Affiliations:** 1 Laboratory of Biostatistics, Clinical Research and Epidemiology and Laboratory of Community Health, Preventive Medicine and Hygiene, Department of Public Health, Faculty of Medicine and Pharmacy, University Mohammed V, Rabat, Morocco; 2 Pedagogy and Research Unit of Public Health, Department of Public Health, Faculty of Medicine and Pharmacy of Rabat, University Mohammed V, Rabat, Morocco; 3 Mohammed V Military Teaching Hospital, Rabat, Morocco; 4 World Health Organization Country Office in Rabat, Rabat, Morocco; 5 Department of Noncommunicable Diseases and Mental Health, World Health Organization Eastern Mediterranean Regional Office, Cairo, Egypt

**Keywords:** prevalence, Eastern Mediterranean region, EMRO, medical students, substance use, alcohol, drug use, drug abuse

## Abstract

**Background:**

The use of psychoactive substances is a growing global public health concern because of its high prevalence and associated risks of morbidity and mortality. In the Eastern Mediterranean Region (EMRO), this issue is particularly pressing among medical students, as it can impact their academic performance, mental health and future professional behaviour.

**Aim:**

This systematic review and meta-analysis aim to determine the prevalence and patterns of psychoactive substance use among medical students in the EMRO region.

**Setting:**

Studies conducted in the EMRO region were included in the analysis.

**Method:**

A systematic search was conducted in PubMed, Scopus and Web of Science, identifying eligible studies. A random-effects model was used to estimate pooled prevalence rates, and meta-regression was performed to assess factors influencing prevalence variation.

**Results:**

Ten studies were included in the study. The pooled prevalence of alcohol consumption among medical students was 9.52% (95% confidence interval [CI]: [4.82–17.93]), which decreased to 5.92% (95% CI: [4.59–7.60]) after removing outliers (*I*^2^ = 81.3%). Meta-regression indicated that studies with sample sizes above 500 reported lower prevalence than smaller studies (β = –1.55, 95% CI: [–2.89; –0.21]). The pooled prevalence of illicit drug use was 9.89% (95% CI: [4.67–19.75]) and 7.78% (95% CI: [3.71–15.58]) after outlier removal.

**Conclusion:**

Substance use among medical students in the EMRO region remains a significant concern.

**Contribution:**

This study highlights the urgent need for preventive strategies to raise awareness and promote healthier behaviours among medical students.

## Introduction

Substance use represents a major public health challenge, impacting the security, safety and development of societies. Addressing it requires a coordinated, multidimensional and multidisciplinary approach. Substance use disorders are linked to various health issues, including mental health conditions, hepatitis, tuberculosis and cardiovascular diseases.^1^ Research also highlights the connection between drug use and criminal activity, with social costs – because of crime, health issues and lost productivity – reaching 2% of gross domestic product (GDP) in some countries.^1^

According to the 2024 World Drug Report, approximately 292 million people (5.6% of those aged between 15 and 64) used drugs in 2022, with cannabis being the most prevalent.^2^ Globally, 64 m individuals suffer from drug use disorders, and injecting drug use remains a significant driver of human immunodeficiency virus (HIV) and viral hepatitis epidemics. Despite this, only 1 in 11 individuals with drug use disorders received treatment in 2022, while the treatment gap in the region exceeds the global average, with just one person in 13 with a substance use disorder receiving the treatment they need.^3^

In the Eastern Mediterranean Region (EMRO), 6.7% of the population aged 15–64 years have used drugs compared with 5.6% globally.^2^ Drug use disorders contribute significantly to disability-adjusted life years (DALYs); age-standardised rates for DALYs from drug use disorders have risen by 20.1% since 1990, surpassing the global increase of 19.0%, with the United Arab Emirates, Libya and the Islamic Republic of Iran recording the highest rates.^4^

Medical studies are considered among the most stressful academic paths because of the high stakes involved, ultimately affecting the lives of the patients whom future physicians will care for. This sort of stress contributes to the harmful use of these substances and causes different disorders.^5^ Understanding the extent and patterns of substance use among medical students is crucial for developing effective interventions and support systems. This knowledge can help in creating strategies to reduce substance use and promote mental health among medical students. In addition, it can guide policy changes in medical education to foster a more supportive and less stressful learning environment.

In the EMRO, a diverse area encompassing countries with varying cultural, economic and healthcare landscapes, this issue is particularly pressing, where the use of psychoactive substances among medical students is a growing concern with significant implications for their academic performance, mental health and future professional behaviour.^6^

Despite this growing concern over substance use among medical students globally, there is a notable gap in the literature concerning this issue within the EMRO. To date, no systematic review has been conducted to comprehensively assess the prevalence and patterns of psychoactive substance use among medical students in this region. This lack of systematic analysis underscores the need for a detailed and structured review specific to the unique sociocultural and economic contexts of EMRO countries.

This systematic review and meta-analysis aim to determine the prevalence and patterns of psychoactive substance use among medical students in this region. By synthesising data from a range of studies, we seek to provide a comprehensive overview of the extent of substance use in this demographic. The findings from this review will guide future research and health promotion strategies as well as the development of targeted interventions and policy measures to address psychoactive substance use among medical students in the region.

## Methods

This review was conducted in accordance with the Preferred Reporting Items for Systematic Reviews and Meta-Analyses (PRISMA) guidelines.^7^

### Search strategy

We screened articles from different databases, including PubMed, Scopus, Web of Science and other sources such as Google Scholar and grey literature. We used the following keywords: ‘Psychotropic Drugs’, ‘Alcohol Drinking’, ‘Prevalence’ and ‘Students, Medical’. These terms were connected using the Boolean operators ‘AND’ and ‘OR’. As for the search on PubMed, equivalent Medical Subject Headings (MeSH) terms were used. After the exclusion of duplicates, articles were filtered by title and abstract and then by full text. The remaining articles were used in a series of papers, including this one about the EMRO region.

### Study selection

Studies were included in this review if they mentioned the prevalence of use of alcohol or any psychoactive drugs among medical students in any of the countries of the EMRO. No language restrictions were applied during the screening process.

The studies that do not give the prevalence of medical students specifically were excluded. We also excluded all narrative or systematic reviews. We used the prevalence of current users in our definition.

### Data extraction

The final selection of studies was assessed to extract relevant data. These data included the authors and year of the study, the country where it was conducted, the sample size, the number of reported cases for alcohol consumption and for drug consumption and the substance used.

### Methodological quality assessment

We used the Joanna Briggs Institute (JBI) critical appraisal checklist for prevalence studies^8^ to assess the quality of our included studies, given that all were prevalence studies. There are nine items rated acceptable (1), unacceptable (0), unclear or not applicable. This assessment was carried out by two independent reviewers, and all doubts were cleared up and resolved by discussion.

### Statistical analysis

Based on relevant publications, we performed a meta-analysis to calculate the overall prevalence of alcohol and illegal drug use among medical students. Cochran’s Q test and the *I*^2^ statistic, which measures variability brought on by heterogeneity rather than chance, were used to assess between-study heterogeneity (*I*^2^ values of 25%, 50% and 75% are generally considered as low, moderate and high, respectively). A fixed-effects model was used when heterogeneity was low to moderate (*I*^2^ < 50%); a random-effects model was chosen in other cases.

Subgroup analyses were conducted based on research quality (poor to medium vs high to excellent), Human Development Index (HDI < 0.70 vs. ≥ 0.70) and sample size (< 500 vs. ≥ 500) in order to further investigate the origins of heterogeneity. The relevance of these variables to the observed heterogeneity was also evaluated using a meta-regression. To investigate the effect of outlier studies on the pooled prevalence estimates, a sensitivity analysis was conducted by excluding them.

Publication bias was assessed by Begg and Egger’s tests and the visual aspect of the funnel plot.

The significance level was set at a *p*-value of less than 0.05. All statistical analyses were performed using R software.

## Review findings

### Flowchart

**FIGURE 1:**
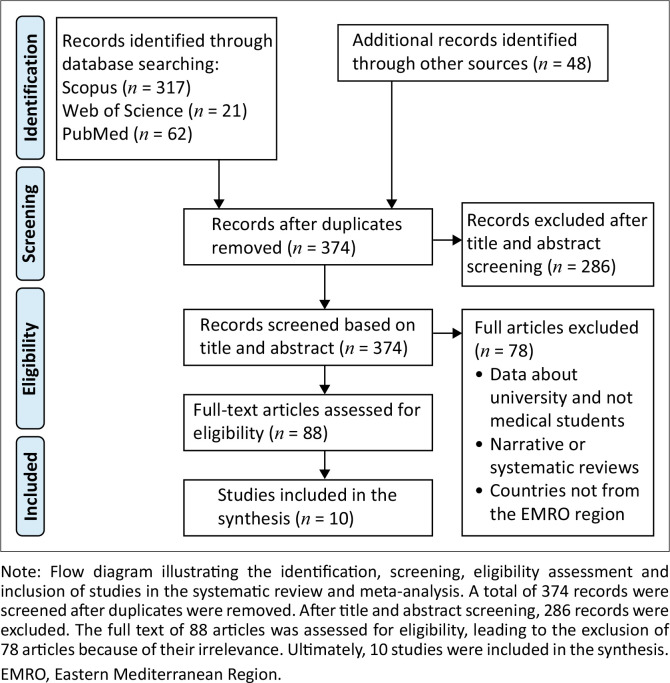
Preferred reporting items for systematic reviews and meta-analyses flowchart of study selection process.

The total number of records from the different sources before sorting was 448. In the end, a total of 10 articles were included in our study, providing the prevalence of use of alcohol or drugs, or both, among medical students in a country from the EMRO (Figure 1).

### Characteristics of included studies

We classified the included studies mentioning the prevalence of use of alcohol (Table 1) based on HDI categories and sample size. Research from Iran, Lebanon and Saudi Arabia, which were classified with an HDI greater than 0.70, generally featured higher total case numbers and larger sample sizes. In contrast, studies from Pakistan and Iraq, which we categorised with an HDI below 0.70, typically had smaller sample sizes and fewer total cases. This classification helps illustrate how research characteristics vary across different human development contexts, highlighting the impact of HDI on study parameters such as case numbers and sample sizes. Although one of the studies in Lebanon^9^ did not focus exclusively on medical students, it was retained in the systematic review because of its partial inclusion of this population. However, given its broader sampling frame, it was excluded from the meta-analysis to maintain comparability across included studies.

**TABLE 1:** Characteristics of studies included in the systematic review and meta-analysis.

Author	Year	Country	Total	Cases of alcohol use	Cases of illegal drug use	Sample size	HDI class	Illicit drugs
Tabrizi et al.^10^	2022	Iran	450	37	90	< 500	> 0.70	Hookah, Tramadol, Ecstasy, Opium
Talih et al.^11^	2018	Lebanon	172	100	60	< 500	> 0.70	Not specified
Pordanjani et al.^12^	2018	Iran	250	12	14	< 500	> 0.70	Ritalin, Tramadol, Ecstasy, Cocaine, Heroin, Methamphetamine, Hashish, Opium
Nawaz et al.^13^	2017	Pakistan	698	24	48	> 500	< 0.70	Cannabis, Amphetamines, Benzodiazepines, Cocaine, Heroin, Opioids, Solvents
Abbasi- Ghahramanloo et al.^14^	2015	Iran	1992	138	57	> 500	> 0.70	Hookah + Others not specified
Jalilian et al.^15^	2015	Iran	355	36	69	< 500	> 0.70	Not specified
Alshammari et al.^16^	2015	Saudi Arabia	975	51	Not specified	> 500	> 0.70	-
Ghandour et al.^9^	2013	Lebanon	570	Not specified	Not specified	> 500	> 0.70	Cannabis, Ecstasy, Cocaine, Opioids
Imran et al.^17^	2011	Pakistan	1299	58	56	> 500	< 0.70	Cannabis, Amphetamines, Benzodiazepines
Ali and Sabir^18^	2009	Iraq	342	81	Not specified	< 500	> 0.70	-

Note: Summary of the studies included in the analysis, detailing the authors, year of publication, country of study, total population surveyed, number of reported cases, sample size category (< 500 or > 500), Human Development Index (HDI) classification (> 0.70 or < 0.70) and the different drugs mentioned by the studies. The studies span multiple countries in the EMRO region with variations in sample sizes and socio-economic contexts. Please see the full reference list of the article Mechita NB, Mountassir AA, Messaoud S, et al. Prevalence of psychoactive substance use among medical students in the EMRO region. J Public Health Africa. 2025;16(1), a1389. https://doi.org/10.4102/jphia.v16i1.1389, for more information.

HDI, Human Development Index.

### Quality assessment results

The methodological quality of the included studies was evaluated using the JBI checklist for prevalence studies.^8^ All studies achieved high scores ranging from 7 to 9 out of a maximum of 9, indicating strong overall quality. Most studies demonstrated appropriate sampling frames, clearly described study settings and subjects and applied valid and consistent methods for identifying and measuring the conditions under investigation. Statistical analyses were generally appropriate, and response rates were mostly adequate. However, a few studies^13,16,18^ had limitations related to the sampling approach, which may introduce a risk of selection bias. Despite these minor concerns, the overall quality of the evidence base was considered robust.

### The prevalence of alcohol consumption

**FIGURE 2:**
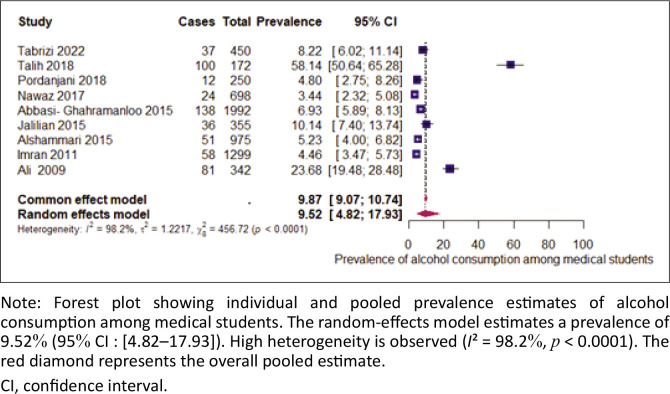
Prevalence of alcohol consumption among medical students in the Eastern Mediterranean Region Region.

The prevalence estimates varied significantly between studies (see Figure 2), with values ranging from 3.44%^13^ to 58.14%.^11^ The pooled prevalence of alcohol consumption among medical students, calculated using a random-effects model because of high heterogeneity (*I*^2^ = 98.3%), was estimated at 9.52%, (95% CI: [4.82–17.93]). After removing outliers, the pooled prevalence was 5.92% with 95% CI: (4.59–7.60), and *I*^2^ = 81.3%.

- The prevalence of alcohol use by income level

The comparison of alcohol consumption prevalence among medical students shows rates of 8.55% in high-income to intermediate-income countries and 11.72% in low-income to intermediate-income countries (Online Appendix 1, Figure 1). High heterogeneity was observed in both groups (*I*^2^ = 95.6% and 99.4%), indicating significant variation across studies. However, the test for subgroup differences (*p* = 0.677) suggests that income level alone does not account for this variation.

### The prevalence of alcohol use by sample size

**FIGURE 3:**
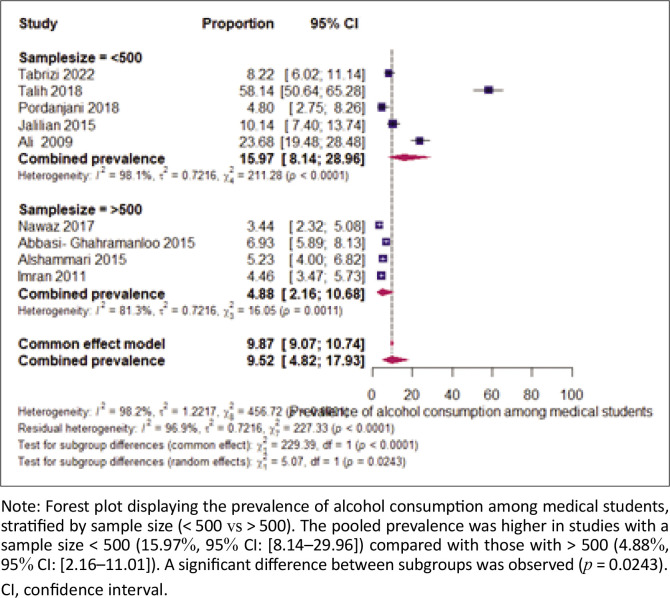
Subgroup analysis of alcohol consumption prevalence by sample size.

The comparison of alcohol consumption prevalence among studies based on sample size shows a higher prevalence in studies with a sample size below 500 (15.97%) compared with those with a sample size above 500 (4.88%) (Figure 3). Heterogeneity was also greater in studies with a sample size below 500 (98.1%) than in those with a sample size above 500 (81.3%). The test for subgroup differences (*p* = 0.024) suggests that sample size may explain the heterogeneity observed in the pooled prevalence.

### The meta-regression

After adjusting for sample size and income level, the analysis revealed that income level was associated with variations in prevalence. Studies with a sample size greater than 500 reported a lower prevalence compared with those with a sample size below 500 (β = –1.55, 95% CI: [–2.89 to –0.21], *p* = 0.023).

Regarding income level, no significant association was found between low-income to intermediate-low-income and high-income to intermediate-high-income groups (β = 0.857, 95% CI: [–0.55 to 2.27], *p* = 0.234).

### The prevalence of illicit drug use among medical students

**FIGURE 4:**
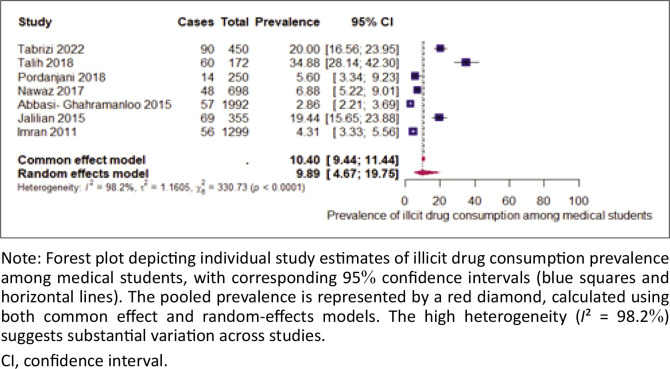
Prevalence of illicit drug consumption among medical students.

The prevalence of illicit drug consumption among medical students across the selected studies ranges from 2.86% to 34.88%. The overall prevalence, based on the random-effects model, was 9.89% (95% CI: [4.67–19.75]), indicating significant variability between studies, as reflected by the high heterogeneity value (*I*^2^ = 98.2%). This suggests considerable differences in drug consumption rates depending on the study contexts (Figure 4).

### The prevalence of illicit drug use among medical students

After removing outliers, the heterogeneity decreased, but it remained high (*I*^2^ = 97.8%). The prevalence of illicit drug consumption varied from 2.86% to 20.00%. The random-effects model, after removing outliers, showed a lower overall prevalence of 7.78%, with a wide confidence interval of 3.71% to 15.58% (Online Appendix 1, Figure 2).

## Implications and recommendations

The prevalence of alcohol consumption among medical students in the EMRO shows variability between the different countries included in this meta-analysis. In this region, cultural, legal and religious factors often discourage or restrict alcohol use, resulting in generally lower prevalence rates (9.53%) compared with regions where alcohol is more socially accepted.^19,20^

However, studies in EMRO reveal significant differences, with reported prevalence rates ranging from as low as 3.44% to as high as 58.14%, as noted in some of the included studies.^11,13^

Many factors can contribute to variability in prevalence estimates, such as:

- The cultural and religious influence: In many EMRO countries, Islamic cultural and religious norms discourage alcohol use, which may reduce prevalence rates. However, the high prevalence reported by the study in Lebanon^11^ can be explained by the fact that the country is one of the few in the region where drinking alcohol is not illegal,^21^ unlike others like Iran and Pakistan.^22,23^ In addition, the diversity of university students and the exposure to Western social norms, Viewing alcohol as a standard part of socialising, can lead to fostering broader acceptance and integration of alcohol use among young adults.^21^
- Sampling and study setting differences: Variations in study design, population sampling and data collection settings contribute to the observed heterogeneity. In our study, the prevalence was higher in studies with a sample size above 500, and the difference was statistically significant.
- Underreporting bias: Social desirability and fear of legal consequences may lead to underreporting of alcohol consumption in some EMRO studies. This can be responsible for lowering reported prevalence rates.
- The income level of the different countries: According to the Global Alcohol Status Reports,^3^ a nation’s income level has a significant impact on the prevalence of alcohol use. Alcohol use is higher in nations with higher incomes. A number of reasons, such as well-established commercial markets that enable availability, sociocultural norms that are more tolerant of drinking and increasing disposable income that improves affordability, are probably responsible for the higher levels of alcohol consumption in high-income nations.^24^ In this study, the prevalence of alcohol use was higher in low-income to intermediate-low-income countries, but the difference was not statistically significant.

Dahlin et al.^25^ reported a higher alcohol consumption among business students of both sexes in comparison with medical students. This difference may be related to the different university cultures: business students are often exposed early to professional networking and social events involving alcohol, while medical students are more frequently confronted with alcohol as a health risk. Since the study, efforts at the Stockholm School of Economics (SSE) have actively addressed this issue, including awareness programmes targeting specific challenges faced by female students and revisions to alcohol policies in SSE-related activities.^25^

In contrast, when examining the use of illicit substances in the EMRO region, the pooled prevalence among medical students was 9.89%. This finding fits within a broader trend, as several studies highlight differences in substance use patterns between medical students and their peers in other academic disciplines. For instance, a survey conducted in Pakistan^26^ revealed that undergraduates who were not studying medicine had a greater frequency of frequent substance misuse (29.4%) than freshmen who were studying medicine (13.4%). Nonetheless, other research indicates that academic and non-academic pressures may make medical university students more susceptible to substance use.^27,28^

Less than half (43.5%) of students in the aforementioned study,^26^ despite the fact that a significant majority (81.7%) were aware of the negative effects of substance use, indicated that they would be willing to stop using drugs if given the opportunity. Encouraging students to participate in healthier alternatives, such as inter-campus competitions, athletics, instructive video screenings and organised discussions on pertinent social topics, may raise their awareness and provide healthy outlets. Additionally, teaching students about the dangers of drug and alcohol use in the classroom might help them become more knowledgeable, develop better coping mechanisms and gain confidence in their ability to make healthier decisions.^29^

Incorporating education on substance abuse into the curriculum can lead to improved outcomes, as suggested by various reports.^30^

## Conclusion

We conclude that while the prevalence of substance use (alcohol and illicit drugs) among medical students in the EMRO region appears lower than in some other regions, it remains a significant concern given its potential impact on students’ well-being and professional development. Institutions should consider strengthening mental health services, offering secure areas for students to receive support and incorporating information about addiction and drug use into medical school curricula. Providing healthy recreational options and encouraging constructive coping mechanisms may also assist in reducing risk. In the end, addressing substance use in this demographic necessitates institutional commitment to student well-being as well as awareness.

## References

[1] Connery HS, McHugh RK, Reilly M, Shin S, Greenfield SF. Substance use disorders in global mental health delivery: Epidemiology, treatment gap, and implementation of evidence-based treatments. Harv Rev Psychiatry. 2020;28(5):316–327. 10.1097/HRP.0000000000000271 32925514 PMC8324330

[2] United Nations Office on Drugs and Crime. United Nations: Office on drugs and crime [homepage on the Internet]. World Drug Report 2024. 2024 [cited 2025 Jan 31]. Available from: //www.unodc.org/unodc/en/data-and-analysis/world-drug-report-2024.html

[3] World Health Organization. Global status report on alcohol and health and treatment of substance use disorders [homepage on the Internet]. Geneva: World Health Organization; 2024 [cited 2025 Jan 31]. Available from: https://iris.who.int/handle/10665/377960

[4] Rostam-Abadi Y, Gholami J, Jobehdar MM, et al. Drug use, drug use disorders, and treatment services in the Eastern Mediterranean region: a systematic review. Lancet Psychiatry. 2023;10(4):282–295. 10.1016/S2215-0366(22)00435-7 36848914

[5] Dumitrascu CI, Mannes PZ, Gamble LJ, Selzer JA. Substance use among physicians and medical students. Med Stud Res J. 2014;3(Winter):26–35.

[6] Ghandour LA, El Sayed DS, Martins SS. Prevalence and patterns of commonly abused psychoactive prescription drugs in a sample of university students from Lebanon: An opportunity for cross-cultural comparisons. Drug Alcohol Depend. 2012;121(1–2):110–117. 10.1016/j.drugalcdep.2011.08.021 21924844 PMC3654541

[7] Page MJ, McKenzie JE, Bossuyt PM, et al. The PRISMA 2020 statement: An updated guideline for reporting systematic reviews. PLOS Med. 2021;18(3):e1003583. 10.1371/journal.pmed.1003583 33780438 PMC8007028

[8] Munn Z, Moola S, Lisy K, Riitano D, Tufanaru C. Methodological guidance for systematic reviews of observational epidemiological studies reporting prevalence and cumulative incidence data. Int J Evid Based Healthc. 2015;13(3):147–153. 10.1097/XEB.0000000000000054 26317388

[9] Ghandour LA, El Sayed DS, Martins SS. Alcohol and illegal drug use behaviors and prescription opioids use: How do nonmedical and medical users compare, and does motive to use really matter? Eur Addict Res. 2013;19(4):202–210. 10.1159/000345445 23391856 PMC3773578

[10] Tabrizi F, Sharafkhani R, Heydari Z, Markani A, Aghziyarat N, Khalkhali H. Estimating the prevalence of high-risk behaviors using network scale-up method in medical university students. J Educ Health Promot. 2022;11(1):356. 10.4103/jehp.jehp_920_21 36618457 PMC9818702

[11] Talih F, Daher M, Daou D, Ajaltouni J. Examining burnout, depression, and attitudes regarding drug use among Lebanese medical students during the 4 years of medical school. Acad Psychiatry. 2018;42(2):288–296. 10.1007/s40596-017-0879-x 29396837

[12] Pordanjani S, Zadeh H, Mousavi M, et al. Prevalence and reasons for psychoactive drugs use among university students of medical sciences in Yazd, Iran. Iran J Psychiatry Behav Sci. 2018;12(1):e9384. 10.5812/ijpbs.9384

[13] Nawaz H, Khan AA, Bukhari S. Use of psychoactive drugs among medical undergraduates in Abbottabad. J Ayub Med Coll Abbottabad JAMC. 2017;29(4):599–603. 29330986

[14] Abbasi-Ghahramanloo A, Fotouhi A, Zeraati H, Rahimi-Movaghar A. Prescription drugs, alcohol, and illicit substance use and their correlations among medical sciences students in Iran. Int J High Risk Behav Addict. 2015;4(1):e21945. 10.5812/ijhrba.21945 25821750 PMC4360541

[15] Jalilian F, Karami Matin B, Ahmadpanah M, et al. Socio-demographic characteristics associated with cigarettes smoking, drug abuse and alcohol drinking among male medical university students in Iran. J Res Health Sci. 2015;15(1):42–46. 25821025

[16] Alshammari FD, Khalifa AM, Kosba AA, et al. Assessment of perception of medical students in regard to links between tobacco or alcohol use and cancer. Asian Pac J Cancer Prev. 2015;16(7):2697–2700. 10.7314/APJCP.2015.16.7.2697 25854349

[17] Imran N, Haider II, Bhatti MR, Sohail A, Zafar M. Prevalence of psychoactive drug use among medical students in Lahore. Ann King Edw Med Univ. 2011;17(4):338–338.

[18] Ali S, Sabir J. Prevalence of alcohol use among medical college students in Hawler Medical University. Zanco J Med Sci. 2009;13:17–23. 10.15218/zjms.2009.004

[19] Bogowicz P, Ferguson J, Gilvarry E, Kamali F, Kaner E, Newbury-Birch D. Alcohol and other substance use among medical and law students at a UK university: Across-sectional questionnaire survey. Postgrad Med J. 2018;94(1109):131–136. 10.1136/postgradmedj-2017-135136 29103016

[20] Gajda M, Sedlaczek K, Szemik S, Kowalska M. Determinants of alcohol consumption among medical students: Results from POLLEK cohort study. Int J Environ Res Public Health. 2021;18(11):5872. 10.3390/ijerph18115872 34070755 PMC8199068

[21] Karam EG, Maalouf WE, Ghandour LA. Alcohol use among university students in Lebanon: Prevalence, trends and covariates. The IDRAC University Substance Use Monitoring Study (1991 and 1999). Drug Alcohol Depend. 2004;76(3):273–286. 10.1016/j.drugalcdep.2004.06.003 15561478

[22] Abbas Khan MA, Akhtar F. Examining the effects of alcohol prohibition Laws in Pakistan on public health. J Pak Med Assoc. 2024;74(12):2155–2159. 10.47391/JPMA.20410 39658987

[23] Chegeni M, Kamel Khodabandeh A, Karamouzian M, et al. Alcohol consumption in Iran: A systematic review and meta-analysis of the literature. Drug Alcohol Rev. 2020;39(5):525–538. 10.1111/dar.13093 32441436

[24] Grittner U, Kuntsche S, Graham K, Bloomfield K. Social inequalities and gender differences in the experience of alcohol-related problems. Alcohol Alcohol Oxf Oxfs. 2012;47:597–605. 10.1093/alcalc/ags040 PMC3417684 22542707

[25] Dahlin M, Nilsson C, Stotzer E, Runeson B. Mental distress, alcohol use and help-seeking among medical and business students: A cross-sectional comparative study. BMC Med Educ. 2011;11(1):92. 10.1186/1472-6920-11-92 22059598 PMC3221703

[26] Mahmud HM, Kalam M, Nawaz A, Khan S, Imam H, Khan OA. Are medical undergraduates more likely to indulge in substance abuse than non-medical undergraduates? A survey from Karachi. J Coll Physicians Surg –Pak. 2014;24(7):515–518. 25052977

[27] Baldwin DC, Hughes PH, Conard SE, Storr CL, Sheehan DV. Substance use among senior medical students: A survey of 23 medical schools. JAMA. 1991;265(16):2074–2078. 10.1001/jama.1991.03460160052028 2013926

[28] Yousafzai AW, Ahmer S, Syed E, et al. Well-being of medical students and their awareness on substance misuse: a cross-sectional survey in Pakistan. Ann Gen Psychiatry. 2009;8:8. 10.1186/1744-859X-8-8 19228374 PMC2660326

[29] Kothari D, Gourevitch MN, Lee JD, et al. Undergraduate medical education in substance abuse: A review of the quality of the literature. Acad Med J Assoc Am Med Coll. 2011;86(1):98–112. 10.1097/ACM.0b013e3181ff92cf PMC3148085 21099395

[30] Muzyk A, Smothers ZPW, Akrobetu D, et al. Substance use disorder education in medical schools: A scoping review. Acad Med J Assoc Am Med Coll. 2019;94(11):1825–1834. 10.1097/ACM.0000000000002883 31663960

